# Agricultural Antibiotics and Human Health

**DOI:** 10.1371/journal.pmed.0020232

**Published:** 2005-07-05

**Authors:** David L Smith, Jonathan Dushoff, J. Glenn Morris

## Abstract

Smith and colleagues discuss evidence suggesting that antibiotic use in agriculture has contributed to antibiotic resistance in the pathogenic bacteria of humans.

Like SARS, Ebola, and other emerging infectious diseases, antibiotic resistance in bacteria may have a zoonotic origin [1]. Evidence suggests that antibiotic use in agriculture has contributed to antibiotic resistance in the pathogenic bacteria of humans, but the chain from cause to effect is long and complicated.

Antibiotic use clearly selects for antibiotic resistance, but how far do these effects extend beyond the population where antibiotics are used? Antibiotics and antibiotic-resistant bacteria (ARB) are found in the air and soil around farms, in surface and ground water, in wild animal populations, and on retail meat and poultry [2–9]. ARB are carried into the kitchen on contaminated meat and poultry, where other foods are cross-contaminated because of common unsafe handling practices [10,11]. Following ingestion, bacteria occasionally survive the formidable but imperfect gastric barrier, and colonize the gut [12].

Patterns of colonization (asymptomatic carriage) and infection (symptomatic carriage) in human populations provide additional evidence that ARB occasionally move from animals to humans [13,14]. The strongest evidence comes from the history of the use of antibiotics for growth promotion in Europe. After first Denmark and then the European Union banned the use of antibiotics for growth promotion, prevalence of resistant bacteria declined in farm animals, in retail meat and poultry, and within the general human population [8,15].

Despite the evidence linking bacterial antibiotic resistance on farms to resistance in humans, the impact of agricultural antibiotic use remains controversial [16–19] and poorly quantified. This is partly because of the complex of population-level processes underlying the between-species (“heterospecific”) and within-species, host-to-host (“horizontal”) spread of ARB. To emerge as human pathogens, new strains of ARB must (1) evolve, originating from mutations or gene transfer; (2) spread, usually horizontally among humans or animals, but occasionally heterospecifically; and (3) cause disease.

All three of these steps are complex and imperfectly understood. The emergence of a new type of resistance is a highly random event, which can't be predicted accurately, and may involve multiple steps that preclude perfect understanding even after the fact. Spread is equally complicated and may obscure the origins of resistance. In some cases, emergence of resistance in one bacterial species is a consequence of the emergence and spread in another species, followed by the transfer of resistance genes from one bacterial species to another. Because of the underlying complexity, mathematical models are necessary to develop theory—a qualitative understanding of the underlying epidemiological processes [20–25]. Theory helps researchers organize facts, identify missing information, design surveillance, and analyze data [26].

## Horizontal Transmission

Theory clearly shows that the impact of agricultural antibiotic use depends on whether resistant bacteria have high, low, or intermediate horizontal transmission rates in human populations [23,24]. The rate of horizontal transmission among humans is determined by the underlying biology of the pathogen, medical antibiotic use, and hospital infection control, but not by agricultural antibiotic use [22]. On the other hand, a farm where multiple antibiotics are used routinely, universally, and in low quantities for growth promotion is likely to be an excellent environment for the evolution of multiple resistance factors, including some variants that might never have evolved in humans. Thus, even very rare transmission resulting from agricultural antibiotics may have a medical impact by introducing new resistant variants to the human population. The epidemiology of spread in the human population dictates how the impact of agricultural antibiotic use should be assessed.

Zoonotic pathogens, such as Campylobacter and Salmonella, are generally regarded as having low horizontal transmission rates in human populations. While resistance in zoonotic infections should be directly attributable to resistance in the zoonotic reservoir, the impact of agricultural antibiotic use remains controversial [18,27–29]. Zoonotic species could acquire resistance genes from human commensal bacteria during the infection process, but this hypothesis is difficult to test.

For pathogens with high horizontal transmission rates, resistant bacteria will spread rapidly once they have emerged, and prevalence will be maintained at a steady state by horizontal transmission. Thus, the impact of subsequent heterospecific transmission is limited (Figure 1). Nevertheless, one or two heterospecific transmission events could be sufficient to cause the appearance of a highly successful ARB genotype in humans, affecting the timing, nature, and extent of spread within the human population [22]. Not only are such events difficult to trace, but their impact is impossible to measure, since there is no way to know what type of resistance would have appeared and with what temporal pattern, if transfers from animals had been prevented.

**Figure 1:**
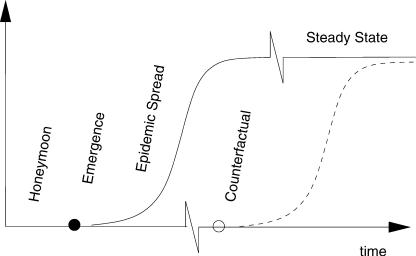
The Emergence and Spread of Antibiotic Resistance in Bacteria with High Horizontal Transmission Rates Emergence and spread begins with a honeymoon period following the approval of a new antibiotic; the honeymoon ends when resistance emerges. Prevalence increases exponentially at first, but it eventually approaches a steady state. The impact of agricultural antibiotic use must be assessed by comparing the observed situation with the counterfactual situation, an imaginary world in which antibiotics were never used in agriculture. The impact of agricultural antibiotic use is, then, the total number of cases of resistance that would not have happened without the use of antibiotics in agriculture. This is approximately the difference between the time of actual emergence and the counterfactual emergence, multiplied by the steady-state prevalence. While we don't know what would have happened in any particular case, we can estimate the likely magnitude of agricultural impacts.

The case where horizontal human transmission rates are intermediate is particularly interesting. If each case in a population generates approximately one new case (a situation we call “quasi-epidemic” transmission), each instance of heterospecific transmission will initiate a long chain of horizontal transmission that eventually burns out. Quasi-epidemic transmission can amplify a relatively low amount of heterospecific transmission and substantially increase prevalence [23–25]. The effect is sustained as long as heterospecific transmission continues. A corollary is that banning agricultural antibiotic use would have maximal benefits if horizontal transmission is quasi-epidemic [24]. Moreover, the effects are most difficult to estimate because both heterospecific and horizontal transmission must be accounted for.

These principles apply to bacteria associated with outpatient antibiotic use and community-acquired infections as well as those that are primarily hospital-acquired. Although quasi-epidemic transmission would seem to be a special case, it may in fact be the rule for many hospital-acquired bacteria because it is the natural endpoint of the interplay between economics and ecology [30]. By spending money on hospital infection control, hospital administrators can reduce nosocomial transmission rates for resistant bacteria. For example, hospitals may screen and isolate patients who are likely to be carriers (i.e., active surveillance) and implement infection-control measures, but this comes at the cost of isolating patients [31]. Total costs are minimized by spending just enough to eliminate (or nearly eliminate) the pathogen; thus, quasi-epidemic transmission is the economic optimum [30].

## The Community as a Reservoir for Resistance

Horizontal transmission is further complicated by population structure, such as the movement of humans through hospitals and long-term care facilities. Medical antibiotic use and horizontal transmission rates are high in hospitals, but this is counterbalanced by short hospital stays. An emerging view for hospital-acquired bacterial infections is that persistent asymptomatic carriage plays a key role in the epidemic of resistance. ARB can asymptomatically colonize a person for years: even if the number of other people infected during a single hospital visit is less than one, this number will exceed one when summed over several hospital visits [25,32,33]. Thus, the ecological reservoir of resistance in the community plays an important role in the increasing frequency of resistance in hospital-acquired infections.

Short hospital visits and long persistence times of ARB in people guarantee that some of the costs associated with failed infection control are passed on to other hospitals—new carriers are frequently discharged from one hospital only to be admitted to another hospital later [30]. Thus, the harm done by these ARB is borne by the whole human population, particularly all of the health-care institutions that serve a single catchment population. In economic terms, the damage caused by the carriage of ARB is a kind of pollution.

By comparing the total number of new carriers generated in the community, the impacts of agricultural antibiotic use on hospitals can be compared directly to the impact hospitals have on each other (Figure 2). The rate of heterospecific transmission is intrinsically difficult to measure directly because the risk of exposure and colonization per meal is very small. Nevertheless, agricultural antibiotic use may generate as many carriers as hospitals for the simple reason that the population experiences many more meals than hospital discharges [34]. When agricultural and nosocomial transmission are equally rare in the population, the latter will be much easier to identify and quantify.

**Figure 2:**
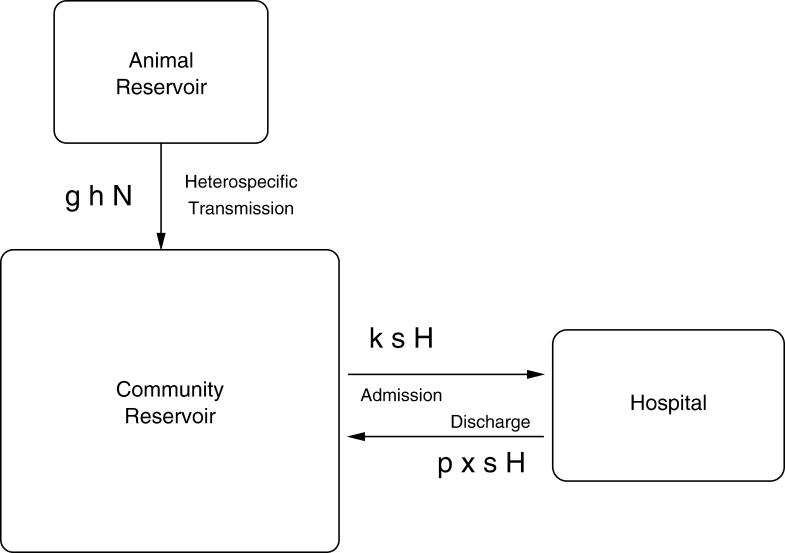
How Large Is the Impact of Antibiotic Use in Agriculture? Comparing the amount of antibiotics used in agriculture with the amount used in medicine means comparing fundamentally different things because they affect the emergence of medically important antibiotic resistance in different ways. For hospital-acquired infections, it is more appropriate to think about ARB carriage in the community as a kind of pollution that flows into hospitals. Thus, the appropriate way to measure impact is by counting how many new carriers are added to the community reservoir from hospital discharges versus from exposure to bacteria that originate on farms. Different formulas describe these processes. To count ARB carriers among hospital discharges, let *x* denote the proportion of patients from a hospital (or other institution) that are colonized on discharge. In some discharged patients, resistant bacteria clear quickly, but a fraction, *p*, become ARB carriers. Some proportion of patients were already carriers at the time of admission, denoted by *k*. Institutions vary by size, *H*, and average length of stay (1/*s*). Thus, the rate that new carriers are discharged from a hospital is given by the formula: *sH(px − k)*. This formula measures the contribution of a hospital to the number of ARB carriers in the community. For example, a hospital with 400 filled beds (*H* = 400 people) serves a US population of about 250,000 people. With a five-day average length of stay (the discharge rate is *s* = 0.2 per patient per day), the hospital discharges about 80 patients each day. If we suppose that 20% of patients acquire resistant bacteria while hospitalized, and one in four of these patients become carriers (*px − k* = 0.05), a hospital would discharge about four persistently colonized people per day—about 1,460 carriers after one year, or approximately 0.58% of its catchment population. A different formula characterizes heterospecific transmission, following exposure to ARB on contaminated food. We let *g* denote the daily per-capita rate that ARB are ingested with a meal. Similarly, we let *h* denote the proportion of those ARB populations that survive the gastric barrier and persistently colonize. The number of new carriers generated in the community by agricultural antibiotic use in a population of size *N* is: *ghN*. For example, if the average person consumes some ARB in 1% of meals (*g* = 0.03 per person per day), followed by colonization with probability one in 2,000 (*h* = 0.0005), agricultural antibiotic use would generate about four new carriers per day in a population of 250,000 people, *N*, approximately the same number as a hospital. The formulas illustrate a general principle: “A large number of people exposed to a small risk may generate many more cases than a small number exposed to a high risk” [34].

## A Natural Experiment: Glycopeptides and Vancomycin-Resistant Enterococci

Is the impact of agricultural antibiotic use on the emergence and spread of ARB in humans large or small relative to medical antibiotic use? Put another way, are farms or hospitals bigger polluters? A large-scale natural experiment was conducted in the United States and several European countries when each adopted different policies on glycopeptide use in animals (avoparcin) and humans (vancomycin) [16,17,35–37]. Many European countries approved avoparcin for animal growth promotion in the 1970s, but the US did not.

In the early 1980s, demand for vancomycin in US hospitals surged because of increasing aminoglycoside resistance among enterococci and methicillin resistance in Staphylococcus aureus. Physicians in US hospitals also used oral vancomycin for some Clostridium difficile infections [37–39]. In the late 1980s and early 1990s, vancomycin-resistant enterococci (VRE) emerged and spread through US health-care systems. In Europe, hospitals used less vancomycin because most enterococci were sensitive to aminoglycosides, and oral vancomycin was seldom used. VRE still emerged and spread through European hospitals, but the problem has been less severe than in the US [40].

A different pattern emerges for community prevalence of VRE. VRE are rarely found outside of hospitals in the US, except for patients who have a prior history of hospitalization. Community prevalence of VRE in the US is typically less than 1%. In contrast, community prevalence of VRE was estimated at 2%–12% in Europe during the late 1990s, including carriage by people with no history of hospitalization [17,41–48]. In other words, the European community reservoir generated by vancomycin use in hospitals and avoparcin use in agriculture was apparently much larger than the US community reservoir generated only by vancomycin use in hospitals.

The prevalence of VRE in the community declined after the EU banned avoparcin [15]. Thus, avoparcin is at least partly responsible for the reservoir of VRE in the European community, but how much of that reservoir came from avoparcin and how much came from hospitals? To weigh the impact, we subtract the community prevalence of VRE in the US (<1%) from the community prevalence of VRE in Europe (>2%). The remainder (>1%) is attributed to avoparcin. This analysis probably underestimates the real impact because vancomycin was used less in European than in US hospitals. Thus, avoparcin use in Europe would appear to be responsible for generating a larger reservoir of VRE in the community than US hospitals. Put another way, the impact of avoparcin use on European hospitals was larger than the impact of US hospitals on one another.

## Conclusion

Despite the evidence that avoparcin use has had a large impact on the emergence and spread of VRE by increasing the reservoir of VRE in the EU, some uncertainty continues to surround the clinical significance of VRE strains of animal origin and of the zoonotic origins of resistance in general. Bacterial strains circulating in hospitalized populations may be genetically distinct from those circulating in the general human population [13,17,49]. Thus, bacterial populations are some combination of zoonotic, quasi-epidemic, and epidemic strains. The complexity of bacterial population biology and genetics makes it practically impossible to trace bacteria (or resistance factors) from the farm to the hospital, or to directly attribute some fraction of new infections to agricultural antibiotic use. Asymptomatic carriage of resistance factors by nonfocal commensal bacteria adds to a general risk of resistance, but transfer of resistance among bacterial species is unpredictable and difficult to quantify. Until more evidence is available, it is prudent and reasonable to consider bacteria with resistance genes a general threat [50–52].

Some part of the controversy over agricultural antibiotic use has been a disagreement about how to weigh evidence and make decisions when the underlying biological processes are complex. In this case, the effects of agricultural antibiotic use on human health remain uncertain, despite extensive investigation, and the effects may be unknowable, unprovable, or immeasurable by the empirical standards of experimental biology. What should be done when complexity makes an important public-health effect intrinsically difficult to measure? What is an appropriate “null hypothesis” or its equivalent? Should the same standards of proof be used in science and science-based policy? Where should the burden of proof fall?

Scientific assessments for policy should summarize the best state of the science, recognizing that the burdens and standards of proof are necessarily softer because of the uncertainty that is introduced by biological complexity. The best decisions weigh the evidence in light of the inherent uncertainty. The EU banned the use of antibiotics for growth promotion, based on the precautionary principle. The use of the precautionary principle was criticized by some as unscientific in this context. In fact, the intrinsic problem of knowability, posed by the biological complexity of the problem, makes the use of precautionary decision making particularly suitable in this arena. The assumption that plausible dangers are negligible, even when it is known that such dangers are constitutively very difficult to measure, may be more unscientific than the use of precaution.

**Summary Points**

- The emergence and spread of ARB is complex and intrinsically difficult to study; mathematical models can help with understanding underlying mechanisms and guiding policy responses.
- Agricultural antibiotic use may generate novel types of ARB that spread to the human population; models can help estimate how much additional disease has been caused by agricultural antibiotic use.
- Transmission of ARB from animal to human populations is particularly difficult to measure, as it is the product of a very high exposure rate to potentially contaminated food, and a very low probability of transmission at any given meal.
- Depending on the assumptions used, the model suggests that transmission from agriculture can have a greater impact on human populations than hospital transmission.
- A comparison of patterns of colonization of VRE in Europe and the United States, which had different patterns of agricultural and hospital antibiotic use, suggests that agricultural antibiotic use can have important quantitative effects on the spread of resistance in the community.

## Abbreviations

ARB: antibiotic-resistant bacteria
VRE: vancomycin-resistant enterococci
